# Case Report: High-grade anterior prostate cancer previously undetected by transrectal biopsy, diagnosed with MRI-US fusion transperineal robotic prostate biopsy

**DOI:** 10.12688/f1000research.109546.1

**Published:** 2022-02-28

**Authors:** Andrian Harsanto, Adistra Imam Satjakoesoemah, Rochani Sumardi, Sahat B.R.E Matondang, Meilania Saraswati

**Affiliations:** 1 Department of Urology, Abdi Waluyo Hospital, Central Jakarta, DKI Jakarta, 10350, Indonesia; 2 Department of Radiology, Abdi Waluyo Hospital, Central Jakarta, DKI Jakarta, 10350, Indonesia; 3 Department of Anatomical Pathology, Dr. Cipto Mangunkusumo Hospital, Central Jakarta, DKI Jakarta, 10430, Indonesia

**Keywords:** anterior prostate cancer, robotic prostate biopsy, targeted biopsy

## Abstract

Seventy percent of anterior prostate cancer cases are diagnosed during rebiopsy. MRI-US fusion transperineal robotic prostate biopsy is an emerging diagnostic method and might be an effective one in diagnosing prostate cancers in difficult sites such as the anterior zone. We report a case of a high grade anterior prostate cancer previously undetected by transrectal biopsy, diagnosed with MRI-US fusion transperineal robotic prostate biopsy. This case report suggests that MRI-US fusion transperineal robotic prostate biopsy might be valuable in diagnosing prostate cancer especially in difficult sites – the anterior region in this case – and might be an imperative diagnostic method in suspicious cases with prior negative biopsy.

## Introduction

MRI-US fusion transperineal robotic prostate biopsy is an emerging diagnostic method for prostate cancer, demonstrating superior accuracy, reduction of tissue trauma, and lower risk for sepsis compared to the conventional systematic biopsy. This method offers great accuracy especially in anterior prostate lesions, which are often missed by conventional systematic biopsy. We present a case of anterior prostate cancer diagnosed using MRI-US fusion transperineal robotic prostate biopsy after prior negative systematic biopsy. ^1^^,^^2^

## Method: MRI-US fusion transperineal robotic prostate biopsy

The robot used was an iSR’obot ^TM^ Mona Lisa transperineal robotic device which utilized a robotic arm mounted to the operation table using a specialized stabilizer (Micro-Touch ^TM^ stabilizer). The system was also connected to a BK Flex Focus 500 ultrasound (BK Medical), with a transrectal probe mounted to the robotic arm to provide a live transrectal ultrasound image during the biopsy. First of all, the patient underwent mpMRI and a radiologist will define the target and boundaries of the prostate using UroFusion (HT) (Biobot Surgical Ltd, Singapore).

The patient entered the operating theatre and was put under general anesthesia to ensure no movement during the procedure and a dwelling urethral catheter was inserted before patient positioning. The patient was placed in lithotomy position while ensuring a secure operating field for the robotic arm movement. The robotic arm then mounted to the operation table and the transrectal probe inserted gently.

The prostate model previously defined by a radiologist was then rendered and the fusion of the prostate model and TRUS images was done using UroBiopsy (Biobot Surgical Ltd, Singapore). Targeted and saturation cores then selected from the fusion model by a urologist. The robotic arm will navigate and position itself according to the assigned target locations and a urologist will obtain a sample using Magnum ^®^ biopsy instrument.

## Case presentation

A 69-year-old Indonesian male was referred to us for an MRI-US fusion transperineal robotic prostate biopsy. He had no history of lower urinary tract symptoms and normal digital rectal examination. His initial PSA result was 7.0 ng/mL and increased to 8.0 ng/mL after a month, he subsequently underwent systematic transrectal prostate biopsy. The patient reported to have experienced fever one day following transrectal biopsy and was treated with oral antibiotic, resulting in symptom resolution. The biopsy result came out negative a month later. He tested his PSA level and it increased to 9.3 ng/mL. He was given oral levofloxacin for a month due to suspected prostatitis. Three months after finishing the regimen, he tested his PSA level and it increased to 17.3 ng/mL.

**Figure 1.**
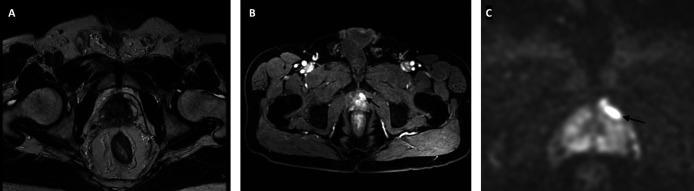
Multiparametric MRI of the prostate (A) T2-weighted axial image shows a homogenous hypointense lesion in the anterior zone of the prostate (B) Dynamic contrast-enhanced image shows contrast enhancement of the lesion; and (C) Diffusion-weighted image of the prostate.

Given the circumstance, multiparametric prostate MRI was done and revealed a 25 cc prostate with a hypointense lesion on T2 at anterior fibromuscular zone showing moderately restricted diffusion and homogenous enhancement after contrast administration, around 1.5 cc in volume, consistent with PIRADS 4 ( Figure 1). The patient was then planned for a targeted rebiopsy and therefore, referred to us. MRI-US fusion transperineal robotic prostate biopsy was done using the iSR’obot ^TM^ Mona Lisa. Five targeted and 14 saturation cores were planned, and all cores were obtained using 2 skin punctures. Intravenous ceftriaxone was given as a prophylactic antibiotic.

**Figure 2.**
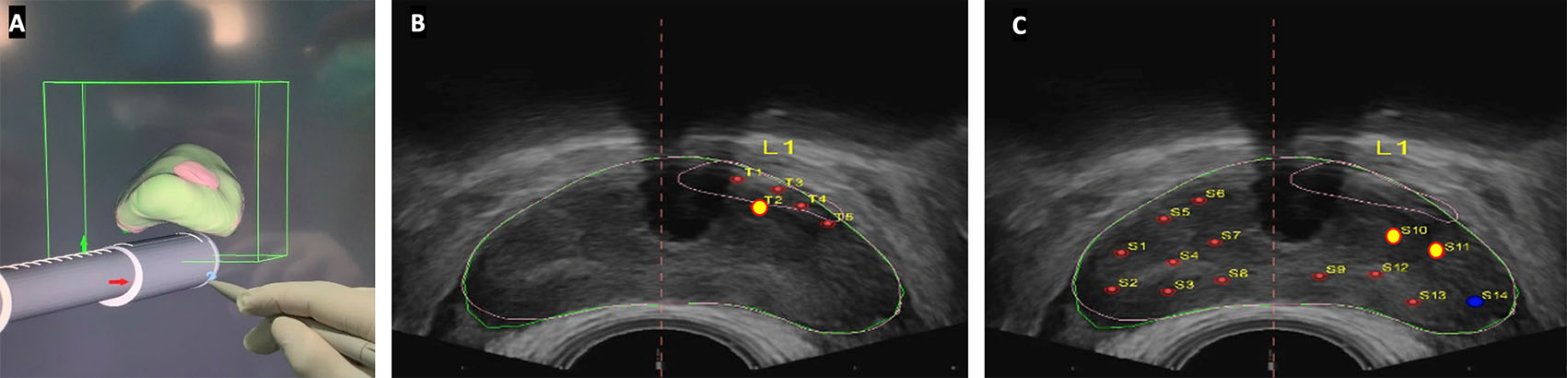
Prostate biopsy targeting (A) 3D view of the target prostate; (B) Targeted plan view (positive lesion marked with a big yellow dot); and (C) Saturation plan view (positive lesions marked with big yellow dots).

The patient was discharged on the same day without any complications. Histopathological examination revealed adenocarcinoma of the prostate, not otherwise specified, Gleason score of 4+4=8, grade group 4, with no perineural invasion. The lesions were found in 1 targeted core (T2) and 2 saturation cores (S10 and S11), the latter were located posterolateral to the targeted lesion ( Figure 2).

## Discussion

A transrectal US-guided prostate biopsy is the gold standard in detection of prostate cancer. ^3^ Unfortunately, its limited accuracy contributes to the misdiagnosis of prostate cancer as one study showed that 70% of anterior prostate cancer cases were diagnosed during rebiopsy. ^4^ Miah, *et al.* stated that the use of robotic prostate biopsy avoids excessive unguided prostate sampling and reduces the number of missed clinically significant prostate cancer (csPCa), this is true especially in our patient with a lesion in the anterior zone. ^3^ Repeat biopsy might not be needed in MRI-US fusion robotic prostate biopsy since samples are taken from a specific and targeted area of the prostate.

In addition to its limited accuracy, transrectal approach is related to a number of post-procedural complications, such as haematospermia (37.4%), haematuria > 1 day (14.5%), rectal bleeding < 2 days (2.2%), prostatitis (1%), fever > 38.5°C (0.8%), epididymitis (0.7%), rectal bleeding > 2 days ± requiring surgical intervention (0.7%), urinary retention (0.2%), and other complications requiring hospitalization (0.3%). ^3^ During his previous transrectal biopsy, the patient experienced fever a day following transrectal biopsy. In contrast, the patient went home the same day without any complications after the robotic biopsy.

In this case, the time of the entire robotic biopsy procedure was 48 min while Kaufmann, *et al.* reported a mean duration of 43 (±6) min for the entire robotic biopsy procedure. Prolonged real-time workflow compared to free-hand transperineal biopsy is one of the disadvantages of robotic prostate biopsy. Another disadvantage is the higher cost of robotic procedure. ^2^ Despite those disadvantages, it is arguable that some patients might benefit from the high accuracy and avoidance of repeat biopsy offered by the MRI-US fusion transperineal robotic prostate biopsy.

While Kaufmann, *et al.* stated that limiting the number of cores might result in efficiency and cost-saving along with a reduced workload burden for pathology department, we still did the biopsy with a high number of cores. ^2^ It was done to ensure a high accuracy and avoidance of repeat biopsy given the circumstance that our patient is highly suspected for prostate cancer with a prior negative biopsy.

Overall, MRI-US fusion transperineal robotic prostate biopsy improves detection in difficult sites while avoiding serious (if any) complications, and further reducing the chance of missing clinically significant prostate cancer, as seen in this patient with a lesion in the anterior zone. ^1^^,^^5^ This also emphasizes the significance of MRI-US fusion transperineal robotic prostate biopsy in diagnosing anterior prostate cancer.

## Conclusion

MRI-US fusion transperineal robotic prostate biopsy is valuable in diagnosing prostate cancer especially in difficult sites – the anterior region in this case – and might be an imperative diagnostic method in suspicious cases with prior negative biopsy.

## Data availability

All data underlying the results are available as part of the article and no additional source data are required.

## Reporting guidelines

Mendeley Data: CARE checklist for High-grade anterior prostate cancer previously undetected by transrectal biopsy, diagnosed with MRI-US fusion transperineal robotic prostate biopsy, http://dx.doi.org/10.17632/4w5rrpv2d7.1.

Data are available under the terms of the Creative Commons Zero “No rights reserved” data waiver (CC0 1.0 Public domain dedication).

## Consent

Written informed consent for publication of clinical details and/or clinical images was obtained from the patient.
