# Supplementary material for: Case Report: High-grade anterior prostate cancer previously undetected by transrectal biopsy, diagnosed with MRI-US fusion transperineal robotic prostate biopsy
Source: F1000Res. 2022 Feb 28;11:247. [Version 1] doi: 10.12688/f1000research.109546.1 (PMC10450259; doi:10.12688/f1000research.109546.1)
Supplement: Peer review history

# Peer review: Reviewer response for version 1

**DOI:** 10.5256/f1000research.121059.r187902

**Published:** 2023-08-24

**Authors:** Christian Wetterauer

**Affiliations:** 1 University Hospital Basel, Spitalstrasse, Basel, Switzerland

This case report presented by Harsanto demonstrates how technically advanced biopsy approaches - like minimally invasive robotic assisted high precision systems - can help to detect clinically relevant prostate cancer otherwise missed by conventional biopsy approaches (especially cancer located in the anterior region). This case also highlights the benefit of MRT imaging prior to biopsy, enabling targeted biopsy of suspicious lesions.

 The patient presented in this case developed infectious complications after transrectal biopsy. Nonetheless, the transrectal approach still represents the most commonly applied approach. However, current guidelines advocate the transperineal approach due to the reduced rate of infectious complications – preferably with minimally invasive systems (like the one presented using two entry points only) in order to minimize side effects and to allow to reduce the application of antibiotic prophylaxis.

 Overall, this report demonstrates how minimally invasive transperineal approaches help to avoid serious complications and at the same time improve detection of cancer located in difficult site by using high precision robotic MRI-US fusion technology.

Are enough details provided of any physical examination and diagnostic tests, treatment given and outcomes?

Yes

Is the case presented with sufficient detail to be useful for other practitioners?

Yes

Is sufficient discussion included of the importance of the findings and their relevance to future understanding of disease processes, diagnosis or treatment?

Yes

Is the background of the case’s history and progression described in sufficient detail?

Yes

Reviewer Expertise:

Prostate cancer diagnostics

I confirm that I have read this submission and believe that I have an appropriate level of expertise to confirm that it is of an acceptable scientific standard.

# Peer review: Reviewer response for version 1

**DOI:** 10.5256/f1000research.121059.r125725

**Published:** 2022-03-22

**Authors:** Isaac Ardianson Deswanto

**Affiliations:** 1 Department of Urology, Faculty of Medicine, University of Indonesia, Jakarta, Indonesia

This article describes a case of high grade anterior prostate cancer that was detected by performing MRI-US fusion transperineal robotic prostate biopsy in a 69 year old patient with prior negative biopsy results from the previous transrectal biopsy.

 The emphasis that is placed on the novelty and superiority of MRI-US fusion transperineal robotic prostate biopsy is well discussed by the authors. It is quite clear that despite the much higher cost, transperineal robotic prostate biopsy offers a high accuracy in detecting prostate cancer especially in detecting cancerous lesions located in the anterior region of the prostate. 

 Overall the case report is well written, concise and very informative. On the other hand however, I would like to suggest the authors provide more details on the previous diagnostic tests of this patients, such as what were the results of the previous MRI examination (if applicable) and how many core biopsy was performed by the previous physician should be mentioned in the case presentation section as well.

Are enough details provided of any physical examination and diagnostic tests, treatment given and outcomes?

Partly

Is the case presented with sufficient detail to be useful for other practitioners?

Yes

Is sufficient discussion included of the importance of the findings and their relevance to future understanding of disease processes, diagnosis or treatment?

Yes

Is the background of the case’s history and progression described in sufficient detail?

Yes

Reviewer Expertise:

General Urology, Andro-urology, endourology

I confirm that I have read this submission and believe that I have an appropriate level of expertise to confirm that it is of an acceptable scientific standard.

# Response

**Published:** 2022-03-23

**Authors:** andrian harsanto

Thank You for the review

 The patient underwent 12-cores systematic transrectal prostate biopsy and had no history of any prior prostate MRI (prior to the one done and mentioned in this case report).
